# Capillary endothelial cell heterogeneity in skeletal muscle of female and male mice

**DOI:** 10.14814/phy2.71025

**Published:** 2026-07-25

**Authors:** Mackenzie E. Charter, Coral L. Murrant

**Affiliations:** ^1^ Department of Human Health Sciences University of Guelph Guelph Ontario Canada

**Keywords:** capillary, endothelial cell, glycocalyx, microvasculature, skeletal muscle

## Abstract

In skeletal muscle, capillaries have been shown to be critical to active hyperemia by sensing skeletal muscle activity and directing blood flow to contracting muscle fibers. There is evidence to suggest that capillary endothelial cell (EC) characteristics may change along the length of a capillary. This longitudinal heterogeneity may have implications on capillary function with respect to their role in active hyperemia. We sought to determine whether EC characteristics differed along the length of a capillary. We perfused 5 female and 6 male CD‐1 mice with fluorescently labeled Wheat Germ Agglutinin (WGA) and GS‐IB4 Isolectin (ISO) which bind to different carbohydrate sites on the EC glycocalyx. We imaged capillaries along their length in fresh whole mounts of the gluteus maximus, diaphragm, soleus, extensor digitorum longus, and cremaster muscles. We observed that WGA labeled capillaries across their entire length while ISO only stained the arteriolar end of capillaries. These findings were observed in all muscles in females and males. These data show that the constituents of the capillary EC glycocalyx are not consistent across the length of a capillary. This capillary heterogeneity may have important implications for the microvascular response during active hyperaemia and blood flow control during muscle contraction.

## 1 INTRODUCTION

In skeletal muscle during exercise, oxygen consumption of contracting muscle fibers can increase 10‐ to 20‐fold requiring a compensatory increase in blood flow up to 100‐fold (for review see Joyner and Casey (2015)) to match metabolic needs in order to maintain proper cellular function and to prevent cellular damage and tissue failure. This active hyperaemia has been shown to be linearly related to a wide range of metabolic demands (for example (Bockman, 1983; Radegran & Saltin, 1998)) and is achieved in the absence of central neural influences (Bockman, 1983; Heinonen et al., 2013), indicating that the coupling of blood flow and metabolic demand occurs locally within the tissue itself. Capillaries have been shown to be mechanistically capable of being integral to the hyperemic response within skeletal muscle. There is a growing body of evidence that establishes the capillaries' ability to be stimulated by contracting skeletal muscle fibers and send vasodilatory signals to upstream arterioles and increase perfusion of the stimulated capillaries that feed the contracting muscle fibers (Berg et al., 1997; Cohen et al., 2000; Cohen & Sarelius, 2002; Lamb et al., 2018; Lamb et al., 2024). Therefore, capillaries can sense the metabolic environment and coordinate the microvascular blood flow response to active cells within a tissue as a critical part of the active hyperemic response.

Capillaries are the smallest level of the microvasculature comprised of a single layer of ECs attached to a basement membrane, whose function has been traditionally treated as uniform across the length of the capillary and whose ECs are treated as homogenous across the length of the vessel. There are, however, indications that capillaries may be comprised of a heterogeneous group of ECs that differ along the length of a single capillary. For example, in rat skeletal muscle, Mrazkova et al. (1986) showed that alkaline phosphatase‐stained segments of capillaries near the arterioles (arteriolar capillaries) and dipeptidylpeptidase IV stained segments of capillaries near the venules (venous capillaries) indicating heterogeneous enzymatic activity between the two sites and between ECs along a capillary. This pattern of staining was not consistent in skeletal muscle from different species (Grim & Carlson, 1990). Additionally, differences between arteriolar and venular capillaries have been observed in angiogenesis where sprouting of capillaries during early wound healing occurred predominantly from the venous capillaries (Phillips et al., 1991) and differential angiogenic responses to VEGF have been observed along the capillary in the retina (Stalmans et al., 2002). Interestingly, in response to endurance training, angiogenesis occurred predominantly on arteriolar capillaries (Suzuki et al., 1997) with training duration (Suzuki et al., 2001) and age‐related changes in venular capillary growth (Cui et al., 2008). Collectively, these studies suggest that capillaries may consist of a heterogeneous population of ECs. Whether EC heterogeneity is a conserved trait of capillaries in skeletal muscle has not been established. Given the potential central role of capillaries in blood flow control in skeletal muscle, it is critical that we establish whether EC heterogeneity exists in skeletal muscle capillaries as a first step in understanding the functional implications of this cellular organization.

Therefore, we sought to explore the potential for EC heterogeneity in capillaries in skeletal muscle by using different lectin stains to visualize capillary ECs in skeletal muscles of female and male mice. Lectins are commonly used to label the vascular EC surface by labeling carbohydrate residues on the EC glycocalyx where different lectins label different elements of the glycocalyx (Sharon & Lis, 2001). We used Isolectin GS‐IB4 from Griffonia Simplicifonia (ISO), which labels α‐D‐galactose carbohydrates and Wheat Germ Agglutinin (WGA), which labels N‐acetylglucosamine and sialic acid carbohydrates, and hypothesized that the glycocalyx of capillary ECs would be heterogeneous along the length of the capillary. We investigated capillaries in gluteus maximus (GM), diaphragm (DIA), soleus (SOL), and extensor digitorum longus (EDL) in females and males as well as the cremaster muscle (CRE) in males to determine whether capillary EC heterogeneity is a common phenotypic feature between skeletal muscles of different fiber types and between sexes.

## 2 METHODS

### 2.1 Animals

All experimental procedures were approved by the University of Guelph Animal Care and Use Committee and were conducted in accordance with the guidelines of the Canadian Council on Animal Care as set out in the *Guide to the Care and Use of Experimental Animals*. A total of eleven CD‐1 mice, five female, six male (age 8–12 weeks; Charles River Ltd., QC, Canada) were used in this study. All mice were housed socially on wood‐chip bedding, had access to food and water ad libitum on a 12:12 h light dark cycle (Envigo 2914, 14% Protein Irradiated Diet). Following all experimental protocols, animals were euthanized with an overdose of sodium pentobarbital (0.26 mg/mL iv to effect).

### 2.2 Fluorescent labeling of capillaries

Two fluorescently labeled lectins were used to investigate capillary EC characteristics: Isolectin GS‐IB4 from Griffonia Simplicifolia Alexa Fluor 568 conjugate (ISO; λ Ex 579 nm; λ Em 603 nm) (Cat# I21412 ThermoFisher Scientific, USA) and Wheat Germ Agglutinin, Alexa Fluor 488 (WGA; λ Ex 495 nm: λ Em 519 nm) (Cat# W11261 ThermoFisher Scientific, USA). ISO was diluted to 166.7 μg/mL in 0.9% saline and WGA was diluted to 1 mg/mL in 0.9% saline. The solutions were divided into 200 μL aliquots and frozen until time of use.

### 2.3 Mouse surgery and capillary labelling

Mice were anesthetized using sodium pentobarbital administered via intraperitoneal injection (75 mg/kg). Depth of anesthesia was monitored by the absence of a withdrawal reflex in response to a toe pinch. Once in the surgical plane, mice underwent a tracheotomy and the insertion of a breathing tube. A catheter was placed in the right jugular vein for further anesthetic administration and dye infusion. 200 μL of ISO and 200 μL of WGA were simultaneously administered to the mouse through the jugular vein catheter. The dyes were allowed to circulate in vivo in the bloodstream for approximately 15 min before the animal was euthanized and skeletal muscles were dissected out.

Four different skeletal muscles were dissected out for imaging of capillaries in females and five in males: gluteus maximus (GM), diaphragm (DIA), extensor digitorum longus (EDL), and soleus (SOL) in females and each of these muscles in males plus the cremaster muscle (CRE). Excised muscles were pinned out in a silicone lined petri‐dish and submerged in room temperature physiological salt solution (PSS) containing (in mMol/L) 131.9 NaCl, 4.7 KCl, 2.0 CaCl_2_, 1.2 MgSO_4_, 30 NaHCO_3_, and 0.3 mg/L tubocurarine hydrochloride pentahydrate aerated with 5% CO_2_ and 95% N_2_ gas to attain a physiological pH (7.35–7.45).

### 2.4 Fluorescent imaging

The fluorescent labelling of the vasculature was detected using an upright Olympus BX51WI microscope (Olympus Canada Inc., Richmond Hill, ON, Canada) using a 10X (na 0.3) objective. Capillaries were visualized using a 100 W mercury arc lamp and 480 ± 20 nm narrow band pass filters for fluorescent excitation of WGA. Emissions were collected using a 500 ± 20 nm long pass filter (Chroma, Brattleboro, VT, USA). Excitation of ISO was achieved with a 560 ± 20 nm narrow band pass filter and emissions were collected using a 580 ± 20 nm long pass filter (Chroma, Brattleboro, VT, USA). Monochromatic images were collected using a cooled CCD camera (CoolSNAP HQ, Roper Scientific, now Teledyne Princeton Instruments, NJ, USA) and RSS Image software on a PC running a Windows 7 operating system. All images were taken under the same collection conditions (i.e., 500 ms capture duration, same mercury arc lamp, etc.).

**FIGURE 1:**
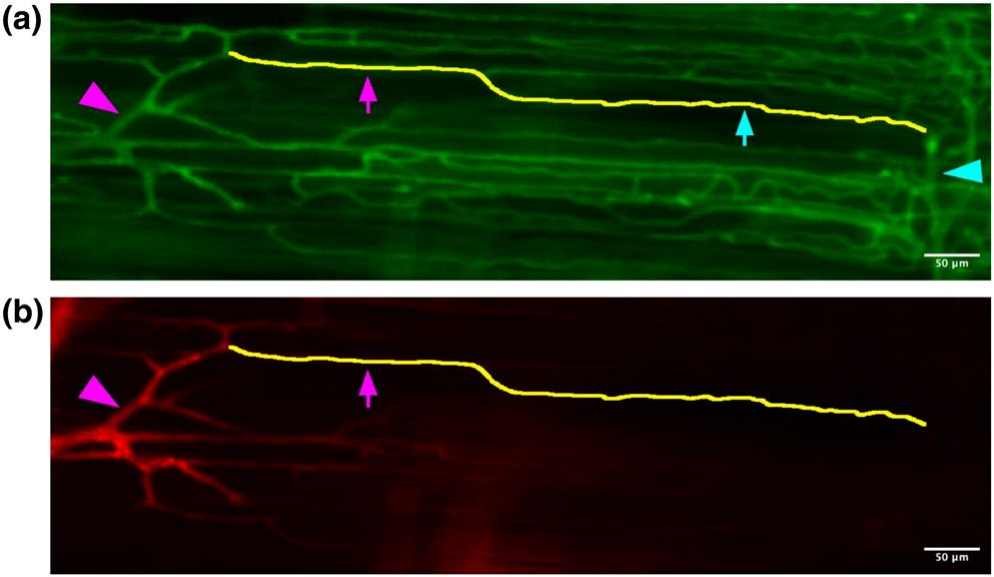
Measurement of brightness of lectin staining across the length of a capillary. Capillary units were identified between the terminal arteriole and venules. A single, in focus capillary that spanned from the terminal arteriole to the venule was imaged using excitation wavelength 488 nm for WGA and 568 nm for ISO. (a) WGA images were colored green and (b) ISO images were colored red. A line ROI was drawn over the in‐focus capillary using the WGA image and brightness was measured at 10 μm increments. The line ROI was then superimposed on the ISO image and brightness measures repeated. Pink arrowheads indicate arterioles and blue arrowheads indicate venules, pink arrows indicate early capillaries stemming from arterioles, blue arrows indicate late capillaries nearing venules.

For each animal, two GM, two EDL, two SOL, one CRE (in males only) and the DIA were removed and multiple areas of each muscle were examined. Images were taken when a full capillary unit could be identified using the WGA label and at least one capillary could be followed continuously from terminal arteriole to collecting venule. Duplicate images of WGA and ISO were taken for each capillary (Figure 1).

### 2.5 Data analysis and statistics

To compare the dyes brightness quantitatively, duplicate images of WGA and ISO stained capillaries were registered using FIJI (https://imagej.net/software/fiji/) to ensure corresponding capillaries in WGA and ISO were overlayed directly on top of each other. Image background was subtracted from the whole image using the FIJI rolling ball algorithm with a rolling ball radius of 30 μm. Following this, a capillary was identified from terminal arteriole to collecting venule in the WGA image (Figure 1a). Brightness was measured within a region of interest (ROI) (line ROI) at the start of the capillary, denoted 0 μm, and subsequent measurements were taken every 10 μm along the capillary over the next 400 μm. The line ROI's were then transposed onto the ISO image and the measurements were repeated (Figure 1b). Brightness data were normalized to a percentage of the initial capillary brightness measurement. Absolute brightness data are represented as area under the curve (AUC) for the full 400 μm segment length, calculated using the FIJI area under the curve function.

Numerical data are reported as mean ± SD while graphical data are reported as mean ± SEM to aid in the clarity of the figures. Differences in WGA staining along the length of a capillary and differences in ISO staining along the length of a capillary were each compared with a one‐way ANOVA. Differences between WGA and ISO staining were compared with a two‐way ANOVA. Sex differences and muscle differences between WGA and ISO staining were compared using a two‐way ANOVA. When the ANOVA identified significant differences, a protected least square difference test was used post hoc to determine specific differences. When comparing muscles within a sex and stain, we used a two‐way ANOVA to determine whether there were differences between muscles. When differences were identified, we used a one‐way ANOVA to determine differences between muscles at each distance measured. When the one‐way ANOVA identified significant differences, a protected least square difference test was used post hoc to determine specific differences at two representative sites along the capillary. Differences were considered significant when *p* < 0.05.

To ensure that dual infusion of dyes did not interfere with the staining pattern of each individual dye, ISO and WGA were infused individually to determine if the staining pattern differed with only one dye present. We found that the staining patterns were similar when dyes were infused individually or together (data not shown).

## 3 RESULTS

The length of capillaries and the total number of capillaries used for measurements in each muscle are shown in Table 1. Between 3 and 13 capillaries were imaged from each muscle.

**TABLE 1:** Average length of capillaries from arteriole to venule in all muscles tested. Length is measured in μm.

	GM		DIA		EDL		SOL		CRE	
	Length	*n*	Length	*n*	Length	*n*	Length	*n*	Length	*n*
Female	450.8 ± 96.9^a^	46	374.1 ± 106.4	42	457.2 ± 132.2^b^	35	358.6 ± 96.5	23		
Male	457.6 ± 143.8	55	362.7 ± 103.1^c^	51	449.9 ± 121.0	30	478.9 ± 147.6^a^	22	448.5 ± 134.0	59

Abbreviation: *n*, number of capillaries counted.

^a^ Indicates where GM in females was different from DIA and SOL in females.

^b^ Indicates where EDL in females was different from DIA and SOL in females.

^c^ Indicates where DIA in males was different from GM, EDL, SOL, and CRE in males.

**FIGURE 2:**
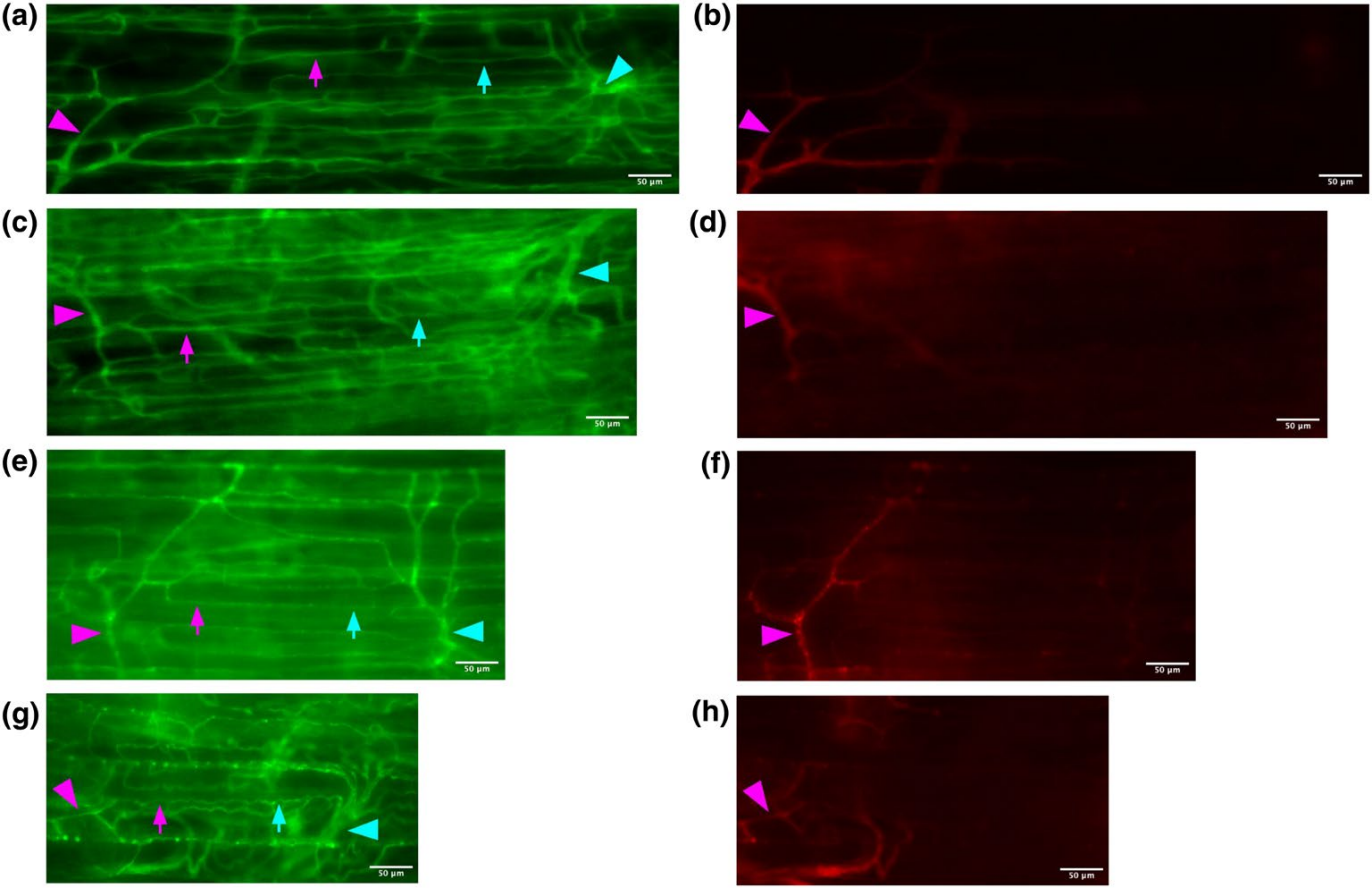
Fluorescent images of skeletal muscle microvasculature in females using WGA (green) and ISO (red). WGA labeled all elements of the microvasculature network including arterioles, capillaries along their length and venules in females in (a) GM (c) DIA (e) EDL (g) SOL. ISO did not label all elements of the microvasculature, labeling arterioles and early portions of the capillaries, but not late capillaries or venules in females in (b) GM (d) DIA (f) EDL (h) SOL. Pink arrowheads indicate arterioles and blue arrowheads indicate venules, pink arrows indicate early capillaries and blue arrows indicate late capillaries.

**FIGURE 3:**
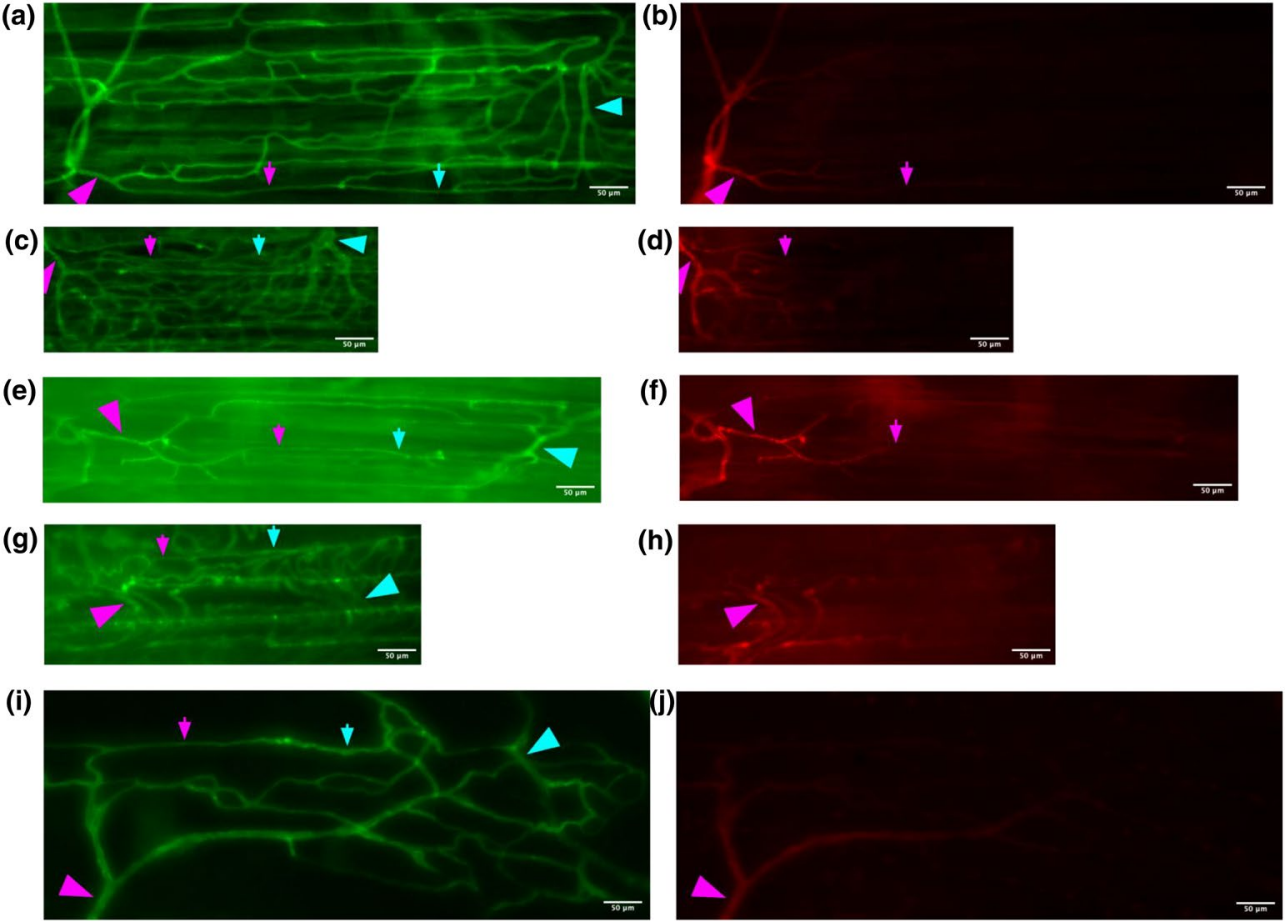
Fluorescent images of skeletal muscle microvasculature in males using WGA (green) and ISO (red). WGA labeled all elements of the microvasculature network including arterioles, capillaries along their length and venules in males in (a) GM, (c) DIA, (e) EDL, (g) SOL, and (i) CRE. ISO did not label all elements of the microvasculature, labeling arterioles and early portions of the capillaries, but not late capillaries or venules in males in (b) GM, (d) DIA, (f) EDL, (h) SOL, and (j) CRE. Pink arrowheads indicate arterioles and blue arrowheads indicate venules, pink arrows indicate early capillaries, and blue arrows indicate late capillaries.

The pattern of staining across the length of the capillary was different between the two lectin stains. WGA staining remained visible across terminal arterioles, the length of a capillary, and collecting venules in all muscles examined (Figures 2a,c,e,g and 3a,c,e,g,i). ISO staining was visible in the terminal arteriole and early capillary but faded significantly and was no longer visible along the late capillary nor the collecting venule (Figures 2b,d,f,h and 3b,d,f,h,j).

### 3.1 WGA and ISO staining in females

**FIGURE 4:**
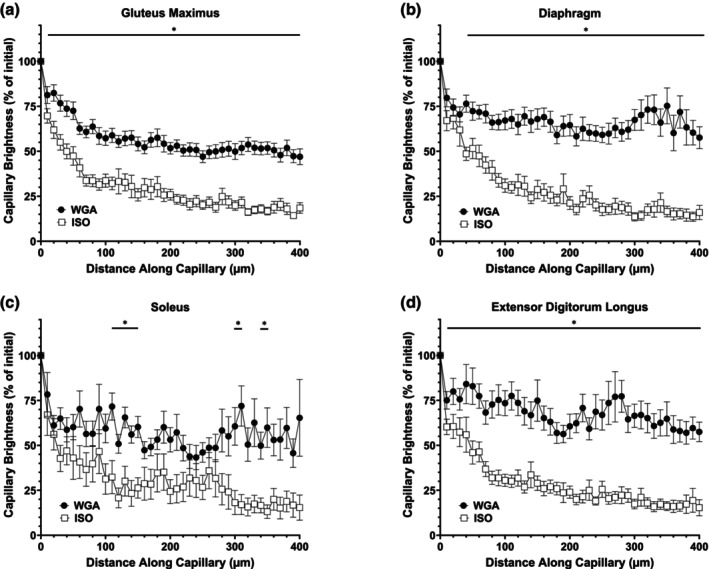
In females, WGA labeled the entire length of the capillary while ISO only labeled arteriolar capillaries. Average normalized brightness along the initial 400 μm of the capillary for WGA (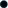) and ISO (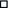) in females in (a) GM (*n* = 46), (b) DIA (*n* = 42), (c) SOL (*n* = 23), and (d) EDL (*n* = 35). Group means over distance for WGA and for ISO were compared using an ANOVA. Differences between WGA and ISO staining were compared with a two‐way ANOVA. When the ANOVA identified significant differences, a protected least squared difference test was used post hoc. Significant differences between WGA and ISO identified by *. Data reported as a percentage of initial capillary brightness. *n* = the number of capillaries observed.

In females, normalized brightness of WGA staining in GM, DIA, SOL, and EDL decreased immediately and significantly along the first 400 μm but remained visible across the length of the capillary (Figure 4). The majority of the decrease in brightness occurred in the first 100 μm (42.7% ± 4.0% in GM, 32.8% ± 5.2% in DIA, 40.6% ± 8.0% in SOL, and 26.5% ± 7.0% in EDL) and by 400 μm fluorescence had decreased by 53.0% ± 4.6% in GM, 42.4% ± 6.0% in DIA, 34.6% ± 21.4% in SOL, and 47.5% ± 5.4% in EDL.

Unlike WGA staining patterns, the normalized brightness of ISO decreased significantly along the length of the capillary and was no longer visible at 400 μm in females (Figure 4). The majority of the decrease in brightness occurred in the first 100 μm (66.3% ± 3.7% in GM, 69.0% ± 3.7% in DIA, 68.7% ± 3.4% in SOL, and 69.5% ± 3.4% in EDL), and by 400 μm fluorescence had decreased by 81.5% ± 2.3% in GM, 96.2% ± 4.0% in DIA, 84.6% ± 7.0% in SOL, and 85.0% ± 4.5% in EDL, respectively.

WGA was significantly brighter than ISO along the length of the capillary in all muscles observed in females (Figure 4). Within the first 50 μm of the capillary WGA staining was significantly brighter than ISO in GM, DIA, and EDL and remained this way for the entire length of the capillary measured. In SOL, while WGA trended brighter than ISO along the length of the capillary, it was only significantly higher at a handful of points.

### 3.2 WGA and ISO staining in males

**FIGURE 5:**
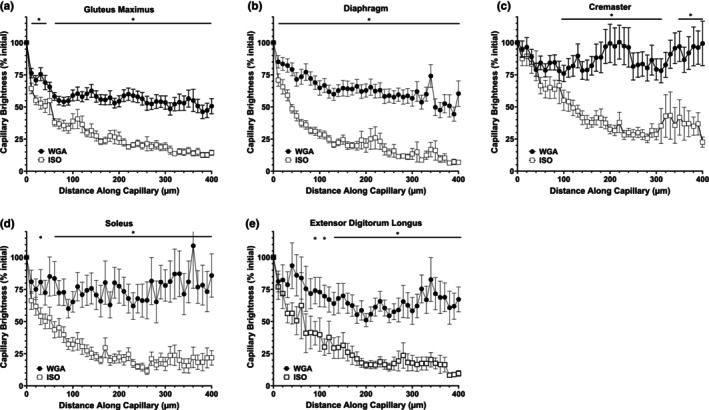
In males, WGA labeled the entire length of the capillary while ISO only labeled arteriolar capillaries. Average normalized brightness along the initial 400 μm of the capillary for WGA (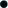) and ISO (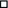) in males in (a) GM (*n* = 55), (b) DIA (*n* = 51), (c) SOL (*n* = 30), (d) EDL (*n* = 22), and (e) CRE (*n* = 59). Group means over distance for WGA and for ISO were compared using an ANOVA. Differences between WGA and ISO staining were compared with a two‐way ANOVA. When the ANOVA identified significant differences, a protected least squared difference test was used post hoc. Significant differences between WGA and ISO identified by *. Data reported as a percentage of initial capillary brightness. *n* = the number of capillaries observed.

In males, WGA staining decreased significantly along the first 400 μm of a capillary in GM, DIA, EDL, and SOL but remained visible across the length of the capillary (Figure 5). WGA normalized brightness decreased over 400 μm by 49.4% ± 5.9% in GM, 37.2% ± 9.9% in DIA, 32.8% ± 9.7% in EDL, 14.1% ± 16.9% in SOL, and 0.7% ± 17.3% in CRE (Figure 5). In GM, DIA, and EDL, the majority of the decrease in brightness of WGA occurred over the first 100 μm (41.0% ± 4.2% in GM, 35.2% ± 5.7% in DIA, and 27.0% ± 11.4% in EDL), while with SOL and CRE WGA brightness did not drop in a similar manner over the first 100 μm.

Unlike WGA staining patterns, the ISO normalized brightness in males decreased significantly along the length of the capillary and was no longer visible by 400 μm (Figure 5). A significant portion of the drop in brightness occurred in the first 100 μm (61.1% ± 5.8% in GM, 71.1% ± 3.2% in DIA, 60.2% ± 13.0% in EDL, 67.6% ± 5.2% in SOL and 44.8% ± 7.9% in CRE) and by 400 μm fluorescence decreased by 85.6% ± 2.0% in GM, 93.0% ± 1.3% in DIA, 90.4% ± 2.3% in EDL, 78.2% ± 5.7% in SOL and 77.4% ± 4.0% in CRE (Figure 5).

WGA was significantly brighter than ISO along the length of the capillary in all muscles observed in males. Within the first 100 μm of the capillary in all muscles observed, WGA staining was significantly brighter than ISO and remained this way for the entire length of the capillary measured (Figure 5).

### 3.3 Male/female comparisons

**FIGURE 6:**
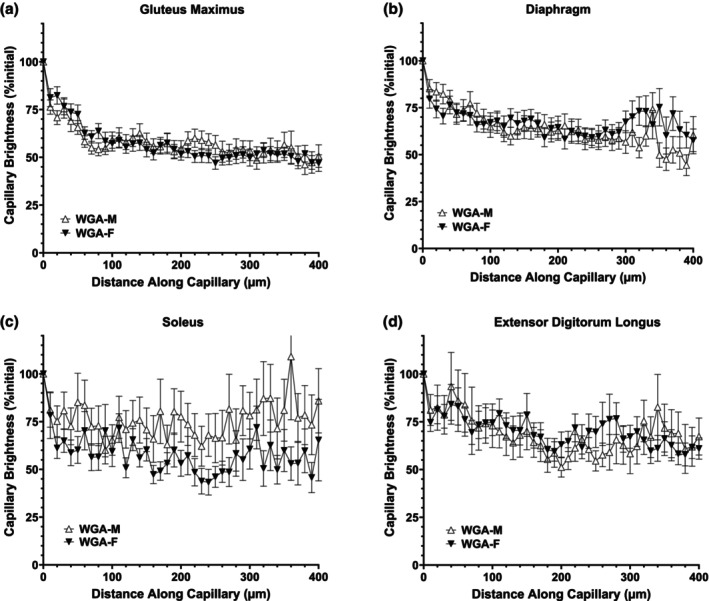
Normalized brightness of WGA did not differ between females and males in any muscle studied. Average normalized brightness along the initial 400 μm of the capillary for WGA in females (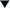) and males (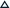) in (a) GM (male *n* = 59, female *n* = 46), (b) DIA (male *n* = 51, female *n* = 42), (c) SOL (male *n* = 22, female *n* = 23), and (d) EDL (male *n* = 30, female *n* = 35). Differences between female and male WGA staining were compared with a two‐way ANOVA. Data reported as a percentage of initial capillary brightness. *n* = the number of capillaries observed.

**FIGURE 7:**
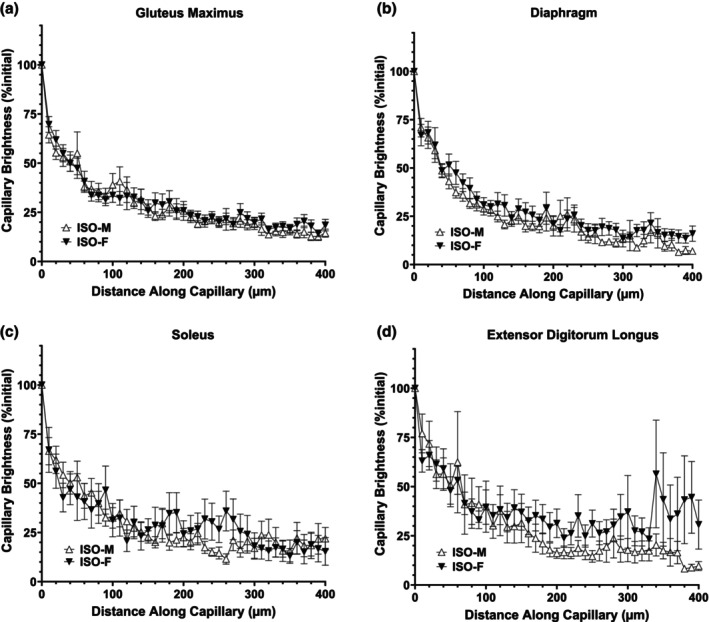
Normalized brightness of ISO did not differ between females and males in any muscle studied. Average normalized brightness along the initial 400 μm of the capillary for ISO in females (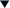) and males (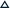) in (a) GM (male *n* = 59, female *n* = 46), (b) DIA (male *n* = 51, female *n* = 42), (c) SOL (male *n* = 22, female *n* = 23), and (d) EDL (male *n* = 30, female *n* = 35). Differences between female and male ISO staining were compared with a two‐way ANOVA. Data are reported as the percentage of initial brightness. *n* = the number of capillaries observed.

Figure 6 compares the normalized brightness of WGA along the first 400 μm of the capillary in female and male muscles. There were no sex differences in WGA staining in any muscle. Figure 7 compares the normalized brightness of ISO along the first 400 μm of the capillary in female and male muscles. There were no sex differences in ISO staining in any muscle.

### 3.4 Muscle comparisons

**FIGURE 8:**
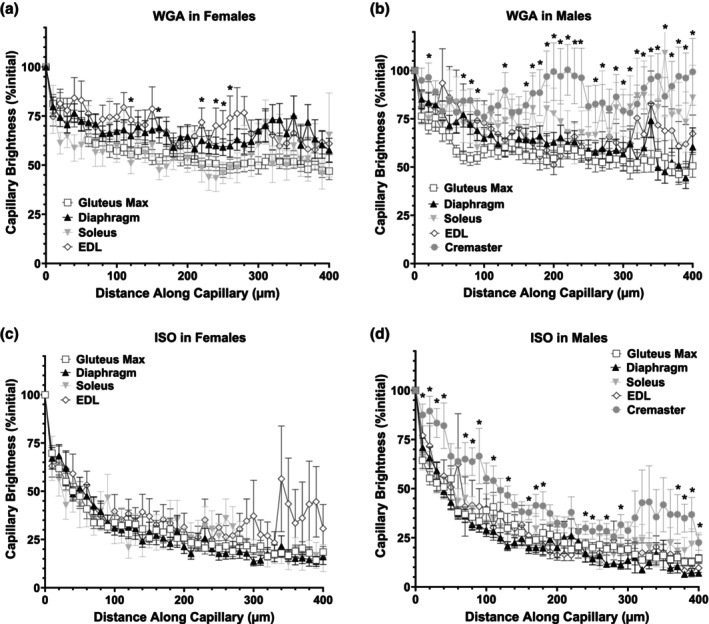
Differences in normalized brightness of WGA and ISO were found between muscles. Average normalized brightness of WGA staining along the initial 400 μm of the capillary in each muscle for (a) females (GM *n* = 46, DIA *n* = 42, EDL *n* = 35, SOL *n* = 23) and (b) males (GM *n* = 59, DIA *n* = 51, EDL *n* = 30, SOL *n* = 22, and CRE *n* = 55). Average normalized brightness of ISO staining along the initial 400 μm of the capillary in each muscle for (c) females and (d) males. Differences between muscles within a sex and a stain were compared using a two‐way ANOVA. When differences were identified we used a one‐way ANOVA to determine differences between muscles at each distance measured. When the one‐way ANOVA identified significant differences, a protected least square difference test was used post hoc to determine specific differences at two representative sites along the capillary. Significant differences between muscles identified by *. Data reported as a percentage of initial capillary brightness. *n* = the number of capillaries observed.

Figure 8 compares the normalized brightness of either WGA or ISO between muscles within a sex, along the first 400 μm of the capillary. In females, there were no differences in ISO staining between muscles (Figure 8c). We identified differences between muscles with WGA staining (Figure 8a). When differences were identified we tested two representative distances along the capillary to determine how the muscles differed. We found at 120 μm EDL was brighter than GM and SOL, and at 240 μm we found that DIA was brighter than SOL. In males, there were differences in staining with both WGA (Figure 8b) and ISO (Figure 8d) between muscles. We identified differences between muscles with WGA staining at 70 μm where DIA, SOL, and CRE were brighter than GM. At 200 μm CRE was brighter than GM, EDL, and DIA, and SOL was brighter than GM and EDL. We identified differences between muscles with ISO staining (Figure 8d) at 90 μm where CRE was brighter than GM and DIA. At 250 μm CRE was brighter than DIA, EDL, and SOL.

### 3.5 Absolute brightness

To estimate whether the total binding site density of a capillary was different between males and females and between muscles we used an area under the curve (AUC) analysis of the absolute brightness values over the 400 μm length of the capillary to represent each muscle and stain as one absolute brightness value. Table 2 shows the absolute brightness represented as AUC in males and females in each muscle. We found that with WGA there were no sex differences in the AUC for absolute brightness between males and females in GM, DIA, and EDL while females had a greater AUC than males in SOL. With ISO there were no sex differences in the AUC for absolute brightness between males and females in GM and EDL while males had a greater AUC than females in DIA and SOL.

**TABLE 2:** Absolute brightness in females and males in all muscles tested. Absolute brightness of fluorescence of WGA and ISO is expressed as area under the curve (AUC) of brightness (in pixels) over the first 400 μm length of the capillary.

	GM		DIA		EDL		SOL		CRE	
	AUC	*n*	AUC	*n*	AUC	*n*	AUC	*n*	AUC	*n*
WGA										
Female	15.0 ± 5.1^d^	46	12.4 ± 4.4	42	13.0 ± 4.8^e^	35	10.3 ± 3.5^a^	23		
Male	15.9 ± 7.0	55	14.4 ± 5.7	51	12.2 ± 4.4	30	7.8 ± 3.0^b^	22	18.3 ± 9.6^c^	59
ISO										
Female	3.3 ± 2.4	46	3.4 ± 2.5^a^	42	3.6 ± 2.5	35	2.5 ± 1.4^a^	23		
Male	4.6 ± 4.8	55	5.3 ± 4.7	51	4.4 ± 3.3	30	4.2 ± 2.9	22	6.9 ± 6.8	59

Abbreviations: AUC, area under the curve measured in pixel*μm/1000; *n*, number of capillaries counted.

^a^ Indicates where females differed significantly from males within the same muscle.

^b^ Indicates where WGA in SOL in males was significantly different from all other WGA in male muscles.

^c^ Indicated where WGA in CRE in males was significantly different from WGA in male SOL, EDL, and DIA.

^d^ Indicates where WGA in GM in females was significantly different from WGA in female SOL and DIA.

^e^ Indicates where WGA in EDL in females was significantly different from WGA in female SOL.

We found that there were no significant differences in ISO AUC for absolute brightness between muscles within males or within females (Table 2). With WGA, there were significant differences in AUC between muscles where, in males, CRE AUC was significantly greater than all other muscles, and all other muscles had a greater AUC than SOL. Muscle differences persisted in females with GM AUC being significantly greater than DIA and SOL, and EDL AUC being significantly greater than SOL.

## 4 DISCUSSION

We sought to determine whether capillary EC characteristics differ along the length of a capillary in skeletal muscles, whether there are EC heterogeneities across muscles of different fiber types, and whether the heterogeneity is similar in males and females. We simultaneously perfused fluorescently labeled ISO and WGA in vivo in CD‐1 mice and examined isolated GM, DIA, SOL, and EDL muscles in males and females, as well as CRE muscle in males, to visualize capillaries and determine whether the EC glycocalyx changes along the length of a capillary. We found that WGA labeled capillaries, allowing for a complete view of the capillary from arteriolar to venular end. ISO labeling diminished significantly along the length of the capillary such that venular capillaries were not visible. This pattern of labeling was observed in all muscles tested and was consistent in both males and females. These data show that capillary EC glycocalyx characteristics change along the length of the capillary, indicating that capillary ECs are heterogeneous along the length of a capillary. This pattern of heterogeneity was consistent across muscles with differing fiber types and consistent in males and females, showing that capillary heterogeneity is a highly conserved trait.

### 4.1 Lectin staining to visualize capillary ECs

We observed ISO binding in arterioles and arteriolar capillaries and that staining of the arteriolar capillaries, but the brightness of the staining diminished over the first 400 μm such that venular capillaries were not visible. This heterogeneity of staining capillary ECs has not been typically observed using ISO and is likely due to the technique used to expose the vascular ECs to ISO. Our methodology of perfusing ISO through the vasculature in vivo exposed the luminal side of the ECs of the vasculature to the dye (Robertson et al., 2015). Methodologies that incubate sectioned tissue or whole‐mount tissues with ISO expose both luminal and basolateral sides of the capillaries to the lectin and observe more uniform staining of all capillaries (Alroy et al., 1987; Barbera‐Guillem et al., 1991; Cebasek et al., 2004; Greene et al., 1990; Hansen‐Smith et al., 1988; Irie & Tavassoli, 1986; Laitinen, 1987; Pena et al., 1981; Peters & Goldstein, 1979; Porter et al., 1990; Thurston et al., 1996). The differences in staining pattern between techniques would indicate that the basolateral side of the capillary EC or the basement membrane (Greene et al., 1990) has a binding site for ISO that is exposed to ISO during cross‐section incubation that is not exposed during perfusion. This differentiation between luminal and abluminal binding has also been observed in hepatic ECs, leading investigators to classify lectins as concordant or discordant for those that bind with the same pattern through incubation and perfusion and those that do not respectively (Barbera‐Guillem et al., 1991). Our results indicate that ISO can be classified as a discordant lectin, and the vessel lumen and basolateral sides have different compositions. Because of this discordant staining characteristic of ISO, perfusion of the vasculature using ISO allowed us to identify longitudinal heterogeneity on the luminal side of ECs in capillaries.

Similar to ISO, perfusion of WGA stained arterioles and arteriolar capillaries and staining also decreased across arteriolar capillaries, but in contrast to ISO, WGA staining remained visible over the entire length of the capillary as well as in venules and veins. This staining pattern of WGA was consistent with other studies using WGA perfusion to visualize the entire microvascular network (Jacobsen et al., 2022; Kataoka et al., 2016) as well as incubation techniques (Alroy et al., 1987; Hausman, 1989; Kirkeby et al., 1991; Pena et al., 1981) indicating WGA is a concordant lectin. While WGA stains across all levels of the microvasculature and makes the entire length of the capillary visible, there was a significant reduction in the amount of staining across the 400 μm length of the capillary segment measured, indicating a heterogeneous binding of WGA across the length of the capillary and an EC heterogeneity across the length of the capillary.

We demonstrated a decrease in ISO and WGA brightness occurred across a 400 μm length of capillary. Arteriolar ECs in rat cremaster, hamster cremaster, and hamster cheek pouch muscles measured 115 ± 3 μm, 124 ± 2 μm, and 141 ± 2 μm in length respectively (Haas & Duling, 1997). If ECs in capillaries are similar in mice and are on average between 115 μm and 141 μm long, we would expect between 2.8 and 3.5 capillary ECs in the first 400 μm of each capillary that we measured. This indicates that the decline in ISO and WGA binding sites happens within the first three ECs. However, since the majority of the decrease in ISO and WGA binding sites occurs within the first 100 μm of the capillary, the decrease in ISO and WGA binding sites could be occurring across the length of a single EC. A better understanding of EC dimensions and morphology of capillary ECs would help with understanding whether there is a heterogeneity across a single EC or across multiple cells in capillaries.

The change in staining pattern along the length of a capillary using WGA and ISO was consistent across each capillary observed in this study. We did not observe one capillary that maintained its ISO staining along the entire capillary and one capillary that did not stain with ISO at all. Such a heterogeneity would imply that one capillary may be optimized for one function while other capillaries are optimized for others. We did not observe this pattern of heterogeneity between capillaries. We observed that each capillary changed their staining patterns along their length in a similar manner, indicating that each capillary may be optimized for different functions at different points along the length of a single capillary, at least with regards to glycocalyx‐dependent functions. Vascular ECs have been shown to be heterogeneous in large vessels, such as the aorta, whereby neighboring ECs respond differently to stimuli (Lee et al., 2018; McCarron et al., 2019). Therefore, it is possible that different patterns of EC heterogeneity exist blood vessels. The specific pattern of heterogeneity we observed along the length of a capillary in skeletal muscle emerged as a result of using markers for the glycocalyx, whereas different patterns of heterogeneity may emerge if other membrane markers or processes are investigated (i.e., adenosine receptors). Interrogation of capillary EC heterogeneity patterns using a variety of cellular markers will help determine the extent of heterogeneity that exists in capillary ECs in skeletal muscle and will help to determine the physiological implications of capillary EC heterogeneity.

### 4.2 Influences of sex and muscle fiber type

The ISO and WGA binding pattern across the capillary was consistent between males and females within each muscle tested. When comparing muscles within a sex, in females, there were no consistent differences in the staining patterns of ISO but a few differences between muscles with WGA staining where SOL tended to be less bright than EDL and DIA but not consistently across the length of the capillary. When comparing muscles within males, CRE stood out as different as both ISO and WGA brightness were greater in CRE than GM, DIA, EDL, and SOL at many points across the first 400 μm of the capillary. Mouse SOL is comprised of approximately 70% type I fiber, EDL is approximately 50% type IIb and 45% type IIa/x, and DIA is approximately 85% type IIa/x (Messa et al., 2019). Although not examined in mice, GM in hamsters predominantly expresses type IIb fibers, and CRE is comprised of between 60% and 80% type IIb fibers (Sarelius et al., 1983; Umek et al., 2023). If fiber type were significantly influencing glycocalyx composition, we would expect CRE and GM to demonstrate similar binding profiles, which we did not; thus, the differences observed in CRE may be driven by a separate factor other than fiber type. In support of this conclusion, we found similarities between the majority of muscles tested indicating that capillary EC heterogeneity does not consistently differ based on muscle fiber type compositions. The lack of sex and muscle differences in both ISO and WGA binding patterns indicates that changes in the EC glycocalyx across the length of a capillary is a conserved physiological characteristic of capillaries in skeletal muscle.

### 4.3 Sex and muscle differences in absolute brightness

Calculation of the AUC for absolute brightness over the initial 400 μm of the capillary was used to estimate total binding site density of N‐acetylglucosamine/sialic acid carbohydrate binding sites for WGA and α‐D‐galactose carbohydrate binding sites for ISO on the glycocalyx. We found that the AUC for WGA was not different between sexes in GM, EDL, and DIA; however, females had a greater AUC than males in SOL. The AUC for absolute brightness for ISO showed no sex differences in GM and EDL; however, males had a greater AUC than females in DIA and SOL. Therefore, there appear to be some sex differences in the amount of N‐acetylglucosamine/sialic acid carbohydrate and α‐D‐galactose carbohydrate binding sites in the glycocalyx between the sexes, with SOL standing out as having differences in both, where females exhibited greater N‐acetylglucosamine/sialic acid carbohydrate binding sites and less α‐D‐galactose carbohydrate binding sites than males.

We also observed differences in the AUC for absolute brightness between muscles within sex. In males, the AUC for absolute brightness for WGA was greater in CRE than all other muscles, and all other muscles had a greater AUC than SOL. Muscle differences persisted in females with the AUC for absolute brightness for WGA was greater in GM than DIA and SOL and where EDL had a greater AUC than SOL. Therefore, SOL had the lowest WGA AUC in both males and females. SOL is approximately 70% type I fibers (Messa et al., 2019), therefore, this finding could represent a relationship between the composition of the glycocalyx (reduced N‐acetylglucosamine and sialic residues) and fiber type. The glycocalyx composition has been shown to differ between tissues and vascular orders (Porter et al., 1990; Thurston et al., 1996) and to change its composition situationally, in response to the tissue environment (Thurston et al., 1996). Our data add to this body of work by showing that the glycocalyx composition may differ between sexes and between muscles.

### 4.4 Prospective physiological significance

Our data clearly show that capillaries are not a uniform vessel segment and, importantly, indicate that capillary ECs within a capillary are not a homogenous set of cells. The physiological relevance of the specific glycocalyx changes identified across the length of a capillary is less clear. Both WGA and ISO bind selectively to different carbohydrate residues within the glycocalyx. WGA selectively binds to N‐acetylglucosaminyl sugar residues and sialic acid (Bains et al., 1992; Bhavanandan & Katlic, 1979). Heparan and keratan sulphate glycosaminoglycan carbohydrate sidechains contain N‐acetylglucosamine as part of their polysaccharide sequences (for review see Couchman and Pataki (2012); Soares da Costa et al. (2017); Moore et al. (2021)). These side chains comprise proteoglycans including glypicans, syndecans, some lecticans, and small leucine‐rich family proteoglycans (SLRPs), making them all potential binding sites for WGA. Further, glycoproteins in the glycocalyx have short carbohydrate side chains capped with sialic acid (for reviews see Cosgun et al. (2020); Foote et al. (2022)), providing further binding sites for WGA on immunoglobulins and selectins. ISO binds to alpha‐D‐galactose carbohydrate residues. Galactose molecules are integrated into glycosaminoglycan sidechains of proteoglycans (Pretorius et al., 2022), which may result in a variety of different sites available for ISO binding. While there has been physiological function associated with specific glycoproteins and proteoglycans, there is no specific physiological function prescribed to the specific carbohydrate side chains that bind WGA and ISO; therefore, the physiological relevance of changes in the carbohydrate side chains in the glycocalyx along the length of the capillary is difficult to ascertain at this time.

Differences in carbohydrate binding sites indicate that the glycocalyx structurally differs along the length of the capillary. In skeletal muscle, the glycocalyx has been assessed to have a thickness of approximately 0.5 μm (Smith et al., 2003; VanTeeffelen et al., 2010; Vink & Duling, 1996), indicating that the glycocalyx extends significantly into the lumen of the approximate 5 μm diameter of a capillary. The glycocalyx‐imposed reduction in diameter of capillaries has been proposed to restrict luminal volume and red blood cell content, slow plasma flow (Desjardins & Duling, 1990; Smith et al., 2003; VanTeeffelen et al., 2010; Vink & Duling, 1996), and act as a significant source of resistance to flow at the capillary level (Pries et al., 1997; VanTeeffelen et al., 2010). Reduction or thinning of the glycocalyx results in an increase in the effective capillary diameter, an increased tube hematocrit (Desjardins & Duling, 1990), reduced resistance (Pries et al., 1997), and allows RBCs closer access to the EC wall (VanTeeffelen et al., 2010). Further, it has been suggested that metabolic stimuli (through muscle contraction) (Klitzman & Duling, 1979) and the vasoactive agonist adenosine (Desjardins & Duling, 1990; Klitzman & Duling, 1979; Platts & Duling, 2004; VanTeeffelen et al., 2005) increase capillary perfusion by decreasing glycocalyx volume. Additionally, vasoactive substances such as bradykinin and sodium nitroprusside (VanTeeffelen et al., 2008) as well as insulin (Eskens et al., 2013) increased capillary tube hematocrit through modifications associated with glycocalyx. Given that the majority of the decrease in PO_2_ from the red blood cell to the skeletal muscle mitochondria occurs from the capillary to the interstitial space (for review see Poole and Musch (2023); Poole and Musch (2023)), any differences in the thickness of the glycocalyx layer may affect oxygen delivery across the capillary wall, and differences across the length of the capillary may contribute to heterogeneity of delivery along the length of the capillary. Thus, structural differences in the glycocalyx across the capillary and modulation of the glycocalyx physiologically may be important factors in the increase in capillary perfusion and change oxygen delivery dynamics during muscle contraction. Our findings of a compositional heterogeneity of the glycocalyx in capillary ECs suggest that any role that the glycocalyx plays in capillary perfusion may not be homogenous along the length of the capillary.

Further, components of the glycocalyx have been shown to be central to the mechanical signal transduction in the process of shear‐stress related NO production by ECs (for review see Power et al. (2024)). Given the potential central role of capillaries in sensing their environment and signaling the upstream arteriolar network during muscle contraction (for review see Murrant et al. (2017)), the glycocalyx may play an important role in blood flow control and signaling at the capillary level during muscle contraction. Therefore, while we cannot ascribe a physiological relevance to our observations of a difference in the glycocalyx from the arteriolar capillary to the venular capillary, a deeper understanding of this heterogeneity is warranted given the potential importance of the glycocalyx in influencing tissue perfusion.

### 4.5 Experimental considerations

Although the fluorescence of the entire length of the capillaries was measured, we report only the first 400 μm of the capillaries from the arteriolar side. This was due to the complexity and density of the microvascular network surrounding the measured capillaries of interest. Towards the venular end of each capillary, there was significant variability in the vascular density as the convergence of all of the capillaries and venules into one collecting venule, and when using WGA to stain the entire vascular network, this venular region tended to become very bright as vessels converged and descended downwards into the tissue. After 400 μm along a capillary, the variability of capillary brightness varied greatly, and it was no longer possible to guarantee that the brightness being measured was an accurate representation of one single capillary. Therefore, capillary brightnesses were reported along the first 400 μm of the capillary. 400 μm on average represented 94.9% ± 11.2% of the total length of the capillary, and all significant binding trends between ISO and WGA were clearly visible within the first 400 μm of the capillary. Therefore, the exclusion of brightness data past 400 μm does not alter our data interpretation.

Skeletal muscles were dissected and pinned out in physiological salt solution at room temperature and may have had to wait to be visualized, conditions that may have led to the generation of hypoxia, which could have affected the staining pattern, but we did not see evidence of this. We would expect that hypoxia would increase over time, but we did not see differences in the pattern of staining when a muscle was imaged first versus when it was imaged last in the series of muscles. Further, muscles were randomized in the order they were viewed with no differences in the pattern of staining; therefore, we do not think that hypoxia had a significant influence on the staining pattern. Further, we confirmed the ISO and WGA capillary staining patterns in the cremaster muscle in situ, using anesthetized mice while observing the exteriorized, blood perfused cremaster using intravital microscopy (Murrant, unpublished data), similar to the methods described by Bagher and Segal (2011) (Bagher & Segal, 2011). Therefore, hypoxic conditions were not a determinant of the pattern of staining on the capillaries.

## 5 CONCLUSIONS

Our data demonstrates a novel characteristic of capillaries in skeletal muscle, that the ECs that comprise a single capillary are heterogeneous along their length. Specifically, we show that components that comprise the glycocalyx differ significantly between the arteriolar capillaries and the venous capillaries in skeletal muscle. And while we did find that the density of specific glycocalyx components was different between sexes and muscles, we found that the pattern of capillary EC heterogeneity was similar in both sexes and each of the five muscles examined indicating that heterogeneity is a conserved physiological trait and a fundamental characteristic of capillaries in skeletal muscle. The physiological relevance of the compositional heterogeneity of the glycocalyx in capillary ECs is unknown at this time but the heterogeneity of capillary ECs raises important considerations with regards to how capillary ECs are organized and may have important implications for capillary function and blood flow control in skeletal muscle. This compositional heterogeneity also raises important considerations for other critical capillary processes such as fluid flux, immune responses, and angiogenesis.

## AUTHOR CONTRIBUTIONS

**Mackenzie E. Charter:** Conceptualization; methodology; investigation; data curation; formal analysis; writing – original draft, review and editing. **Coral L. Murrant:** Conceptualization; investigation; data curation; formal analysis; writing – original draft, review and editing; funding acquisition; project administration; supervision.

## FUNDING INFORMATION

This study was supported by the NSERC Canada RGPIN‐2025‐004177.

## CONFLICT OF INTEREST STATEMENT

None to declare.

## ETHICS STATEMENT

All procedures were approved by the Animal Care Committee at the University of Guelph and were performed in accordance with the Canadian Council of Animal Care as set out in the Guide to the Care and Use of Experimental Animals.

## INFORMED CONSENT

Not required.

## Data Availability

Data will be made available upon reasonable request.
